# Complete Genome Sequence of a Colistin-Susceptible Salmonella enterica Serovar Minnesota Strain Harboring *mcr-9* on an IncHI2/IncHI2A Plasmid, Isolated from Chicken Meat

**DOI:** 10.1128/MRA.00826-21

**Published:** 2021-11-11

**Authors:** Khaloud O. Alzahrani, Elaf A. Alshdokhi, Mohammed I. Mujallad, Fahad M. Al-Reshoodi, Ashwaq S. Alhamed, Abdulrahman A. Alswaji, Maha A. Alzayer, Liliane Okdah, Majed F. Alghoribi, Sulaiman M. Alajel

**Affiliations:** a Reference Laboratory for Microbiology, Research and Laboratories Sector, Saudi Food and Drug Authority, Riyadh, Saudi Arabia; b Infectious Diseases Research Department, King Abdullah International Medical Research Center, Riyadh, Saudi Arabia; c King Saud bin Abdulaziz University for Health Sciences, Riyadh, Saudi Arabia; University of Arizona

## Abstract

The recent emergence and dissemination of mobilized colistin resistance (*mcr*) genes have triggered extensive concerns globally. Here, we report the complete genome sequence of a colistin-susceptible Salmonella enterica serotype Minnesota strain (named SA18578), belonging to sequence type 548 (ST548) and carrying the *mcr-9* gene on an IncHI2/IncHI2A plasmid, that was isolated from chicken meat in Saudi Arabia in 2020.

## ANNOUNCEMENT

The mobile colistin resistance (*mcr-9*) gene was first detected in Salmonella enterica serotype Typhimurium, which highlighted the need for rigorous monitoring of the potential spread of this new gene (1). Here, we determined the complete genome sequence of an *mcr-9*-carrying Salmonella enterica serotype Minnesota strain that was isolated from chicken meat.

This strain was isolated and confirmed to belong to Salmonella enterica serotype Minnesota based on the International Organization for Standardization guideline ISO 6579-1:2017 (2). Resistance to antibiotics was determined using standard broth microdilution and CLSI guidelines (3). MIC results showed that SA18578 was resistant to amoxicillin-clavulanic acid, ampicillin, gentamicin, sulfisoxazole, and tetracycline; interestingly, SA18578 exhibited susceptibility to colistin (≤1 mg/liter).

For DNA extraction, this strain was grown overnight in Oxoid nutrient agar at 37°C, and DNA was extracted using the QIAamp DNA minikit following the manufacturer’s instructions (Qiagen). The short reads for this strain were generated by a MiSeq system (Illumina, CA) with a MiSeq v3 kit using 2 × 300-bp paired-end chemistry. Raw data were demultiplexed using Illumina’s bcl2fastq tool (version 2.20) and checked for quality using the FastQC tool (version 0.11.9) (4). The long reads for this strain were generated on a MinION sequencer (Oxford Nanopore Technologies) using the ligation sequencing kit SQK-LSK109. Default parameters were used for all software unless otherwise specified. The MinION raw data were base called using Guppy (version 4.3.4), which is integrated into MinKNOW (version 21.02.5), followed by filtering of reads with a minimum Q score of 7 and a minimum read length of 1,000 bp. A total of 595,286 short reads and 55,129 long reads were combined to obtain the complete genome sequence of the SA18578 isolate using the EToKi pipeline (Enterobase Tool Kit) (version 1.0) (5), generating 5 contigs with an average sequencing coverage depth of 40×. The NCBI Prokaryotic Genome Annotation Pipeline (PGAP) (version 5.2) (6) was used for genome annotation.

The genome of SA18578 consisted of one 4,826,311-bp chromosome, with a GC content of 52%, and four plasmids, designated pSA18578_MCR9_1 (IncHI2/IncHI2A; 352,141 bp [GC content of 48%]), pSA18578_2 (IncA/C2; 129,660 bp [GC content of 51%]), pSA18578_3 (Col440I; 3,228 bp [GC content of 48%]), and pSA18578_4 (3,383 bp [GC content of 55%]).

Multilocus sequence typing (MLST), plasmid replicon typing, and determination of antibiotic resistance genes were performed with ABRicate (version 0.9.8) (https://github.com/tseemann/abricate) using MLST (version 2.0.4) (7), PlasmidFinder (version 1.3) (8), and ResFinder (version 2.1) (9, 10). *In silico* MLST analysis indicated that the SA18578 strain belonged to sequence type 548 (ST548). Plasmid pSA18578_MCR-9_1 carried the colistin resistance *mcr-9* gene and belonged to the incompatibility group IncHI2/IncHI2A. In addition to *mcr-9*, the resistance genes *bla*_TEM-1B_ and *aac(3)-IId* were identified on this plasmid. Plasmid pSA18578_3 carried one resistance gene (*qnrB19*), and no resistance genes were identified on pSA18578_2 and pSA18578_4. Further resistance genes were detected on the chromosome, including *sul2*, *tet*(A), *ant(3″)-Ia*, and *bla*_CMY-2_, as well as a point mutation in *parC* (T57S).

Based on our results, it appears that the presence of the *mcr-9* gene is actually not associated with colistin resistance. A few studies investigated the role of the *mcr-9* gene in colistin resistance and concluded that this gene was neither inducible nor expressed under normal conditions (1, 11, 12). Therefore, further studies are necessary to determine the impact of the *mcr-9* gene on colistin susceptibility.

### Data availability.

All sequence data were deposited in NCBI GenBank under BioProject accession number PRJNA751432 and SRA accession numbers SRR16071937 (short reads [Illumina]) and SRR16069012 (long reads [Oxford Nanopore Technologies]). The complete genome assemblies of the Salmonella enterica serovar Minnesota SA18578 isolate can be found under GenBank accession number CP080513.1 for the chromosome, accession number CP080514.1 for pSA18578_MCR9_1, accession number CP080515.1 for pSA18578_2, accession number CP080516.1 for pSA18578_3, and accession number CP080517.1 for pSA18578_4.
